# Rapid profiling of *Plasmodium* parasites from genome sequences to assist malaria control

**DOI:** 10.1186/s13073-023-01247-7

**Published:** 2023-11-10

**Authors:** Jody E. Phelan, Anna Turkiewicz, Emilia Manko, Joseph Thorpe, Leen N. Vanheer, Marga van de Vegte-Bolmer, Nguyen Thi Hong Ngoc, Nguyen Thi Huong Binh, Nguyen Quang Thieu, Jesse Gitaka, Debbie Nolder, Khalid B. Beshir, Jamille G. Dombrowski, Silvia Maria Di Santi, Teun Bousema, Colin J. Sutherland, Susana Campino, Taane G. Clark

**Affiliations:** 1 Faculty of Infectious and Tropical Diseases, London School of Hygiene & Tropical Medicine (LSHTM), London, WC1E 7HT UK; 2 Department of Medical Microbiology and Radboud Center for Infectious Diseases, Radboud University Medical Center, University of Nijmegen, Nijmegen, The Netherlands; 3 Molecular Biology Department, Parasitology and Entomology, Vietnam National Institute of Malariology, Hanoi, Vietnam; 4 Directorate of Research and Innovation, Mount Kenya University, Gen. Kago Rd, Thika, Kenya; 5 UK Health Security Agency Malaria Reference Laboratory, LSHTM, London, WC1E 7HT UK; 6 Department of Parasitology, Institute of Biomedical Sciences, Univ. of São Paulo, São Paulo, Brazil; 7 School of Medicine, Instituto de Medicina Tropical, University of São Paulo, São Paulo, Brazil; 8 Faculty of Epidemiology and Population Health, LSHTM, London, WC1E 7HT UK

**Keywords:** Drug resistance, Malaria, *Plasmodium* parasites, Genomics, Diagnostics, Whole genome sequencing

## Abstract

**Background:**

Malaria continues to be a major threat to global public health. Whole genome sequencing (WGS) of the underlying *Plasmodium* parasites has provided insights into the genomic epidemiology of malaria. Genome sequencing is rapidly gaining traction as a diagnostic and surveillance tool for clinical settings, where the profiling of co-infections, identification of imported malaria parasites, and detection of drug resistance are crucial for infection control and disease elimination. To support this informatically, we have developed the *Malaria-Profiler* tool, which rapidly (within minutes) predicts *Plasmodium* species, geographical source, and resistance to antimalarial drugs directly from WGS data.

**Results:**

The online and command line versions of *Malaria-Profiler* detect ~ 250 markers from genome sequences covering *Plasmodium* speciation, likely geographical source, and resistance to chloroquine, sulfadoxine-pyrimethamine (SP), and other anti-malarial drugs for *P. falciparum*, but also providing mutations for orthologous resistance genes in other species. The predictive performance of the mutation library was assessed using 9321 clinical isolates with WGS and geographical data, with most being single-species infections (*P. falciparum* 7152/7462, *P. vivax* 1502/1661, *P. knowlesi* 143/151, *P. malariae* 18/18, *P. ovale* ssp. 5/5), but co-infections were identified (456/9321; 4.8%). The accuracy of the predicted geographical profiles was high to both continental (96.1%) and regional levels (94.6%). For *P. falciparum*, markers were identified for resistance to chloroquine (49.2%; regional range: 24.5% to 100%), sulfadoxine (83.3%; 35.4– 90.5%), pyrimethamine (85.4%; 80.0–100%) and combined SP (77.4%). Markers associated with the partial resistance of artemisinin were found in WGS from isolates sourced from Southeast Asia (30.6%).

**Conclusions:**

*Malaria-Profiler* is a user-friendly tool that can rapidly and accurately predict the geographical regional source and anti-malarial drug resistance profiles across large numbers of samples with WGS data. The software is flexible with modifiable bioinformatic pipelines. For example, it is possible to select the sequencing platform, display specific variants, and customise the format of outputs. With the increasing application of next-generation sequencing platforms on *Plasmodium* DNA, *Malaria-Profiler* has the potential to be integrated into point-of-care and surveillance settings, thereby assisting malaria control. *Malaria-Profiler* is available online (bioinformatics.lshtm.ac.uk/malaria-profiler) and as standalone software (https://github.com/jodyphelan/malaria-profiler).

**Supplementary Information:**

The online version contains supplementary material available at 10.1186/s13073-023-01247-7.

## Background

Malaria is a life-threatening disease caused by *Plasmodium* parasites that are transmitted to humans by infected female *Anopheles* mosquitoes [1]. There were 247 million cases of malaria and 619 thousand deaths in 2021 alone, with the vast majority affecting children and pregnant women in Sub-Saharan Africa [1]. There are six parasite species that cause malaria in humans (*P. falciparum*, *P. vivax*, *P. ovale* ssp., *P. malariae*, *P. knowlesi*; genome sizes 23–36 Mbp). *P. falciparum* is the deadliest malaria parasite and the most prevalent on the African continent. *P. vivax* is the most geographically widespread malaria parasite [2], found in Europe, Asia, South America, and Africa due to its adaptation for temperate climatic conditions. Whilst zoonotic *P. knowlesi* is found primarily in Southeast Asia due to the presence of the macaque population, which acts as a reservoir for the parasite. *P. ovale* ssp. and *P. malariae* malaria cases have been predominantly reported in Africa and can occur with co-infections with *P. falciparum,* potentially affecting elimination strategies that target prevalent species [3].

Malaria treatment is guided by the knowledge of the infecting *Plasmodium* species and clinical severity. There are currently fourteen medicines for the treatment of malaria and four for preventative treatment listed by the World Health Organization (WHO) [4]. Global efforts to control and eliminate malaria are hampered by the emergence of *P. falciparum* parasites resistant to antimalarial drugs. There is a high prevalence of chloroquine and sulfadoxine-pyrimethamine (SP) resistance across continents [5], and partial resistance or slow parasite clearance to artemisinin, used in current treatment combinations, spreading in Southeast Asia [6], with some recent cases appearing in Africa [7]. Similarly, *P. vivax* isolates resistant to chloroquine have been reported in parts of Asia and South America [2].

Prompt malaria diagnosis either by microscopy or rapid diagnostic tests (RDTs) is recommended by the WHO for all patients with suspected malaria before they are given treatment [4]. Early and accurate diagnosis is essential both for effective management of the disease and for strong malaria surveillance. Measures targeting the treatment of persistent malaria infections, such as *P. ovale* ssp. and *P. vivax* with dormant liver stages and *P. malariae* with possible latent blood infections, will need to comprise all human malaria species. Neither microscopy nor RDTs can detect low-density malaria infections, common in both low and high transmission settings, but nucleic acid amplification tests (NAATs) such as Polymerase chain reaction (PCR), real-time PCR (rt-PCR), loop-mediated isothermal amplification (LAMP), and quantitative nucleic acid sequence-based amplification (QT-NASBA) assays can overcome this limitation. The 18S ribosomal RNA gene has unique sequences that enable the identification of all six malaria species infecting humans and is therefore commonly targeted for amplification. Similarly, the mitochondrial genome (6 kbp) has species-specific markers, and has the added advantage of being present in high copy numbers in *Plasmodium* cells [8]. A number of studies have revealed *P. falciparum* genetic markers linked to antimalarial drugs, such as chloroquine, SP and artemisinin [9–11], which are being included within NAATS, but the underlying mechanisms for *P. vivax* chloroquine resistance are unclear [2].

The increasing accessibility of advanced high throughput technologies that are cost-effective and with low sequencing error rates, can inform clinical decision making and tracking of infections. Recently, whole genome sequencing (WGS) has gained traction as a diagnostic tool for infections, with the ability to determine strain types of pathogens, characterise transmission patterns, and identify markers linked to antimicrobial resistance [12]. Portable platforms, such as Oxford Nanopore Technology (ONT), are facilitating the real-time generation of sequencing data in the field and clinic. Such platforms can also be used to sequence large numbers of amplicons (~ 500 bp) that cover candidate genes, across many samples, leading to a high throughput low-cost diagnostic tool that can capture new variants in targeted loci [13, 14]. However, one of the main challenges in performing WGS or amplicon-based sequencing studies for clinical malaria parasites is the difficulty in obtaining sufficient high-quality parasite DNA from infected individuals. This difficulty is due to low parasitaemias in infections and human DNA “contamination”. However, recently a selective whole genome amplification (SWGA) strategy has been used to sequence *P. falciparum* [15]*, P. vivax* [2, 16], *P. knowlesi* [17] and *P. malariae* [18] genomes from non-filtered blood and from dried blood spots of clinical samples, leading to the characterisation of single nucleotide polymorphisms (SNPs) and insertions and deletions (indels) for population genomic analyses. More generally, genomic diversity studies using WGS from *P. falciparum*, *P. knowlesi* and *P. vivax* endemic field isolates have provided significant insights into the structure and ancestry of the geographical-based parasite populations, intra- and inter-population genomic diversity, and led to the development of molecular barcodes to determine the geographical source of infections [8, 17, 19–21]. Furthermore, population genetic analyses have identified genomic regions under selective pressure, some in drug resistance-associated genes [2, 15, 22–24].

As the generation of WGS and amplicon-based sequencing data for *Plasmodium* parasites continues to increase at a swift pace, including from the portable ONT platform, there is a need for informatics tools for researchers and applied bioinformaticians to rapidly analyse WGS data. Such tools are needed to obtain profiles of (co-)infections and drug resistance markers, thereby supporting clinical decision-making. Further, by additionally identifying likely geographical origin (e.g. country), it could reveal imported parasites, thereby supporting surveillance decision-making too. By monitoring the changes in informative mutations temporally, it will allow an assessment of transmission patterns and the effectiveness of infection control activities. Here, building on the core library used in a similar software for tuberculosis (“TB-Profiler” [25, 26]), we describe the *Malaria-profiler* standalone and web-based tool, with accompanying dashboard interfaces, for rapid profiling of *Plasmodium* parasites species, and characterising genetic variants for follow-up studies.

## Implementation

### Profiling mutation library

**Fig. 1:**
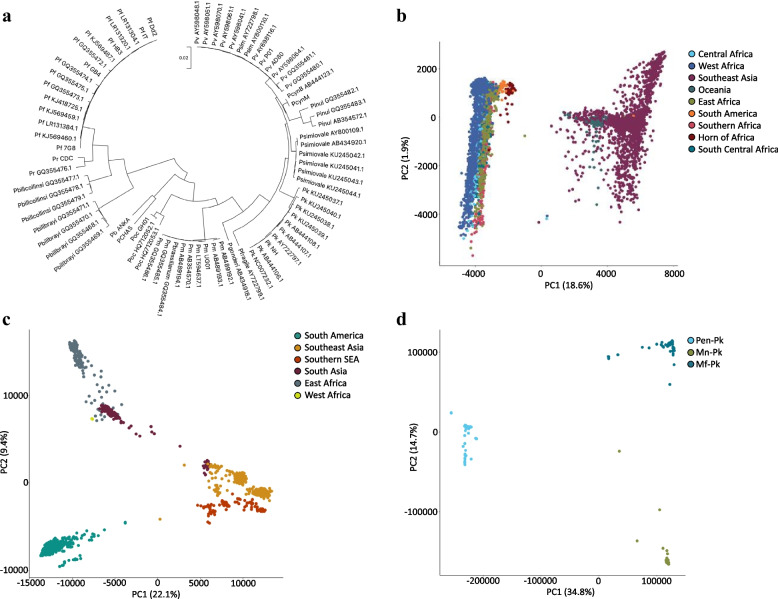
Population structure of *Plasmodium.* **a** Circular maximum likelihood tree of 51 human and 24 non-human *Plasmodium* isolates using mitochondrial sequences shows perfect clustering of species as expected. This indicates the presence of a species-specific sequence which is exploited in the k-mer-based speciation function. Pf *P. falciparum*, Pv *P. vivax*, Pk *P. knowlesi*, Pcyn *P. cynomolgi*, Pm *P. malariae*, Poc *P. ovale curtesi.* **b** *P. falciparum* principal component analysis showing clustering by geographic region specifically separation between Southeast Asia and Oceania and Africa. **c** *P. vivax* principal component analysis showing clustering by geographic region. **d** *P. knowlesi* principal component analysis showing clustering by region (Peninsular (Pen-Pk) vs. Borneo Malaysia), and within Borneo based on host (*Macaca fascularis* (Mf-Pk) and *Macaca nemestrina* (Mn-Pk))

The mutation library consists of ~ 100 mitochondrion markers for speciation of *P. falciparum*, *P. vivax*, *P. malariae*, *P. knowlesi* and *P. ovale* ssp*.* (20 per species; Table 1), which also differentiate human from non-human affecting *Plasmodium* species*.* In brief, alignments of 75 mitochondrial genomes (51 human and 24 non-human *Plasmodium*; Fig. 1a) were used to construct a maximum likelihood phylogenetic tree. By annotating the tree branches with ancestral mutations [26], it was possible to define k-mers (31 bp) using *kmc* software [27], from which 20 SNPs exclusive to each human species were determined. Using the mitochondrial genome has the advantage of ~ 20 more copies than the nuclear genome in cells [8]. In addition, we included a set of established markers (*n* = 137) that differentiate geographical regions for *P. falciparum* (61; Eastern, Western and Horn of Africa, Southeast Asia, South America, Oceania), *P. vivax* (56; East Africa, South Asia, Southeast Asia, Southern Southeast Asia, South America) and *P. knowlesi* (20; Non-Borneo (Peninsular); Borneo – *Macaca fascularis* (Borneo-Mf), Borneo – *Macaca nemestrina* (Borneo-Mn)) [8, 17, 19, 20] (Table 2). In brief, these barcoding markers have been previously determined using the population differentiation F_ST_ statistic, and identifying scores of one, which indicate that the SNP allele is fixed in the region of interest and not present outside that location. Lastly, known drug resistance mutations (*n* = 37) across *P. falciparum* candidate genes [15] were also included in the library (Table 3) as well as genetic variants in putative drug-associated loci reported for other malaria species (e.g. orthologues of *Pfcrt*, *Pfdhfr*, *Pfdhps*, *Pfkelch13* and *Pfmdr1*) [2, 18, 21]. The mutation libraries are available and hosted on the GitHub open-source site, with versioning capability (https://github.com/jodyphelan/malaria-db). Future changes in the species, geolocation and drug resistance mutation libraries can be discussed, tracked, and visualised as part of the GitHub hosting. This method of hosting also enables multiple users and developers across the malaria genomics community to contribute to the project.

**Table 1:** Predictions of *Plasmodium* species using *Malaria-Profiler* library (*n* = 9312)

Source label	No. markers*	*Pf*	*Pv*	*Pk*	*Pm/Pbr*	*Poc/Pow*	Other	Mixed**	Total
*Pf*	20	7152	11	1	-	-	-	298	7462
*Pv/Psim*	20	-	1502	-	-	-	9	150	1661
*Pk*	20	-	-	143	-	-	-	8	151
*Pm/Pbr*	20	-	-	-	18	-	-	-	18
*Poc/Pow*	20	-	-	-	-	5	-	-	5
Other***	-	-	-	-	-	-	24	-	24
Total	100	7152	1513	144	18	5	24	456	9312

^*^ https://github.com/jodyphelan/malaria-db

^**^ Mixed co-infections with source

^***^ Non-human, including *P. inui*, *P. cynomologi*, *P. reichenowi* (see Fig. 1a)

*Pf*, *P. falciparum*; *Pv*, *P. vivax*; *Po*, *P. ovale* ssp.; *Pm*, *P. malariae*; *Pbr*, *P. brasilianum*; *Pk*,* P. knowlesi*

**Table 2:** Accuracy of the *Malaria-Profiler* library for geographical predictions

Parasite	No. markers*	Region	No. samples	No. predicted	% Sensitivity***	% Specificity***
*P. falciparum*	8	Africa**	4181	4138	99.0	98.3
*P. falciparum*	10	Eastern Africa	1153	1051	91.2	99.9
*P. falciparum*	10	Western Africa	3028	2964	97.9	99.1
*P. falciparum*	10	Horn of Africa	16	16	100	100
*P. falciparum*	8	Oceania	144	132	91.7	100
*P. falciparum*	6	South America	48	45	93.8	100
*P. falciparum*	9	Southeast Asia	2741	2626	95.8	99.9
*P. vivax*	9	East Africa	158	154	97.5	100
*P. vivax*	11	South America	455	382	84.0	100
*P. vivax*	36	Asia	889	799	89.9	99.8
*P. vivax*	14	South Asia	195	157	80.5	99.9
*P. vivax*	13	Southeast Asia (SEA)	537	488	91.0	99.6
*P. vivax*	9	Southern SEA	157	141	89.8	99.3
*P. knowlesi*	7	Borneo-Mf	53	52	98.1	98.9
*P. knowlesi*	6	Borneo-Mn	40	39	97.5	100
*P. knowlesi*	7	Peninsular	50	50	100	100

^*^https://github.com/jodyphelan/malaria-db

^**^Without Horn of Africa

^***^Assuming that the meta data location is the gold standard

*Peninsular*, Non-Borneo; *Borneo-Mf*, Borneo – *Macaca fascularis*; *Borneo-Mn*, Borneo – *Macaca nemestrina*; *N*, sample size

**Table 3:** Drug resistance based on known mutations in P. falciparum*

Region	N**	Chloroquine	Pyrimethamine	Sulphadoxine	Artemisinin
East Africa	1153	0.245	0.932	0.905	0
West Africa	3028	0.372	0.800	0.839	0
Horn of Africa	16	1	1	0.875	0
Oceania	144	0.667	0.910	0.306	0
South America	48	0.854	0.917	0.354	0
Southeast Asia	2741	0.711	0.876	0.831	0.306
Overall	7152	0.492	0.854	0.833	0.118

^*^World Health Organization mutations in https://github.com/jodyphelan/malaria-db

^**^Excludes 22 laboratory strains without a known source location

### In silico* profiling*

The *Malaria-Profiler* tool for the in silico analysis of species, geolocation and drug-resistant mutations was developed using the Python language (v3.8) with the pathogen-profiler library [12] and well-established bioinformatic tools such as *trimmomatic *[28], *BWA *[29] and S*AMtools *[30]. The pipeline can be customised (Additional file 1: Fig. S1), but in its default mode, reads are trimmed using *trimmomatic (*parameters: LEADING:3 TRAILING:3 SLIDINGWINDOW:4:20 MINLEN:36) then mapped to the appropriate *Plasmodium* reference (e.g. *P. falciparum* 3D7, *P. vivax* PvPO1) using *bwa* (with default parameters). The raw data can be in an Illumina or ONT format (Additional file 1: Fig. S2). With the default settings, variants are called using *freebayes *[31] (parameters: -F 0.05) and annotated using *snpEff *[32] (parameters: -noLog -noStats), with the processing parallelised using GNU parallel [33]. Annotated variants are compared to the list of mutations in the *Malaria-Profiler* libraries. Variants can be filtered using coverage depth, allele frequency and per-strand depth parameters that can be set by the user. Additionally, other variant calling tools can be used instead of *freebayes* with *bcftools *[30] and *gatk *[34] also implemented. A minimum depth of tenfold coverage to call variants is set as the default (consistent with [5, 12]), but this can be changed by the user. Positions below this cut-off will be recorded and presented in the final report. The *Malaria-Profiler* pipeline calculates the proportion of the reads supporting each allele and reports this information, which can serve as a proxy for multi-infections. The *Malaria-Profiler* pipeline is available on GitHub (from https://github.com/jodyphelan/malaria-profiler) and can be installed through the *bioconda* channel [35]. *Malaria-Profiler* report outputs are written in *json*, *txt* and *pdf* formats, with options to collate data into multi-sample reports in a dashboard (Additional file 1: Fig. S2).

### Sequencing data and variants

To test the *Malaria-Profiler* tool, a dataset of 9321 strains was collated from Illumina WGS raw data in the public domain (see https://www.ebi.ac.uk/ena). This database includes *P. falciparum* (*n* = 7462; https://www.malariagen.net/apps/pf6 [36]; PRJEB2136, PRJEB2143, PRJEB4348, PRJEB4410, PRJEB4580, PRJEB4589, PRJEB4611, PRJEB4725, PRJEB5045, PRJNA108699 and PRJNA51255), *P. vivax* (*n* = 1661, https://www.malariagen.net/data/open-dataset-plasmodium-vivax-v4.0; PRJEB10888, PRJEB2136, PRJEB2140, PRJEB4409, PRJEB4410, PRJEB44419, PRJEB4580, PRJEB56411, PRJNA175266, PRJNA240366-240531, PRJNA271480, PRJNA284437, PRJNA295233, PRJNA420510, PRJNA432819, PRJNA603279, PRJNA643698, PRJNA65119, PRJNA655141, PRJNA67065, PRJNA67237, and PRJNA67239) [2, 21], *P. knowlesi* (*n* = 151; PRJEB10288, PRJEB1405, PRJEB23813, PRJEB28192, PRJEB33025, and PRJNA294104) [17, 20], *P. malariae* (*n* = 18; PRJEB33837) [18] and *P. ovale* (*n* = 5; PRJEB51041) [18]. Meta data, including the geographical site of sampling, was available from the same sources (e.g. www.malariagen.net/resources/open-data-resources). In addition, the mitochondrion reference genomes were obtained from GenBank for the neglected non-human malaria parasites (*n* = 24; e.g. *P. cynomologi*, *P. inui*, *P. reichenowi* and *P. simiovale*) were also included in the analysis. Alignments of mitochondrial genomes to the species library allow for the identification of primary *Plasmodium* infection and potential co-infections. Species were assigned if half of the 20 specific markers were identified in the data. For intra-species analysis, genome-wide SNPs and indels were called using established bioinformatic pipelines [2, 15, 17, 18]. In brief, the raw Illumina WGS data (fastQ format) were aligned to their respective reference genomes using *BWA-mem* software (default parameters). SNPs and short indels were called using the S*AMtools* and *GATK* software suites (see [19]). For ONT data, a similar pipeline was adopted, except sequence alignment was performed using *minimap2 *[37] software.

### Using genomic data to inform on *Plasmodium* parasite speciation and geographical clustering

A maximum likelihood phylogenetic tree for *Plasmodium* species was constructed using *RAxML-NG* (v 0.9.0; 1000 bootstraps) software applied to mitochondrial genomes (*n* = 75; 5592 nucleotides), which were aligned using *MUSCLE* software [38] and filtered with the *Gblocks* tool [39] (default settings). The optimal substitution model of nucleotide or amino acid evolution for phylogenetic construction was determined by *MEGAX* software [40]. Parasite clustering within species (e.g. *P. falciparum*, *P. vivax*; total *n* = 9321), which is typically geographically based, was explored by performing a principal component analysis (PCA) on the isolates using pairwise Manhattan distances based on biallelic SNPs.

### Malaria-Profiler performance

To test the performance of the library, the WGS raw data for the 9321 strains were processed through the *Malaria-Profiler* pipeline to predict species, geolocation, and resistance status (for *P. falciparum*). The predictions were then compared to primary *Plasmodium* species and geographical recorded meta information (see www.malariagen.net/resources/open-data-resources), which were assumed to be the gold standard, and thereby allowed the calculation of the predictive accuracy of the *Malaria-Profiler* library. Phenotypic drug resistance status was not available for most isolates. Samples identified by *Malaria-Profiler* with potential co-infections were also analysed with *Centrifuge* software [41] to confirm the main *Plasmodium* species. When applying *Centrifuge*, the threshold for potential co-infection was based on the whole genome abundance (minimum 5%) and samples with > 1 *Plasmodium* species exceeding the threshold were assigned as mixed. To demonstrate the utility of WGS in the clinic, processed DNA (see [42] for protocols) from two isolates (isolate1, isolate2) sourced from two malaria patients at the Radboud University Medical Center were sequenced on the ONT MinION platform (v10) at The Applied Genomics Centre, LSHTM (accession numbers ERR11254081 and ERR11254083).

## Results

### Species prediction

Using the mitochondrion alignments of 51 human and 24 non-human *Plasmodium* parasites, a phylogenetic analysis revealed clustering by species (Fig. 1a), as well as the robustness of the species-level barcoding markers used within the *Malaria-Profiler* library. Across the 9321 isolates with WGS data, the *Malaria-Profiler* tool predicted the labelled primary species in almost all samples (9300/9321; 99.8%). Mixed co-infections were also detected (456/9321; 4.9%), with *P. falciparum* (298; 63.4%) and *P. vivax* (150; 32.9%) being the dominant parasites (Table 1), and most co-infections were supported by a parallel analysis using *Centrifuge* software (*P. falciparum* 165/298, 55.4%; *P. vivax* 116/150, 77.3%). Discrepancies arise due to *Centrifuge* software excluding genomes with very minor frequencies (< 5%). The 24 non-human related *Plasmodium* mitochondrion sequences were also processed by the tool, leading to the predicted (and expected) absence of any of the six human-affecting *Plasmodium* species (Table 1).

### Geographical predictions

The geographical-based population structure of *P. falciparum*, *P. vivax*, and *P. knowlesi* was confirmed using a principal component analysis of SNPs which revealed clustering by geographic region (Fig. 1b–d). Using the geographical barcodes on isolates with recorded location (*n* = 8775), the *Malaria-profiler* tool predictions were accurate to continental (96.1%) and regional (94.6%) levels (Table 2). The best performance was for *P. knowlesi* (141/143; 98.6%), known to display high variability between clusters [20]. The accuracy for *P. falciparum* predictions was high (6834/7130; 95.8%), across all regions (> 91%). The accuracy for *P. vivax* was lower (1322/1502; 88.0%), especially for the South Asia region (80.5%), due to high similarity between neighbouring countries across regions.

### Genotypic drug resistance

**Fig. 2:**
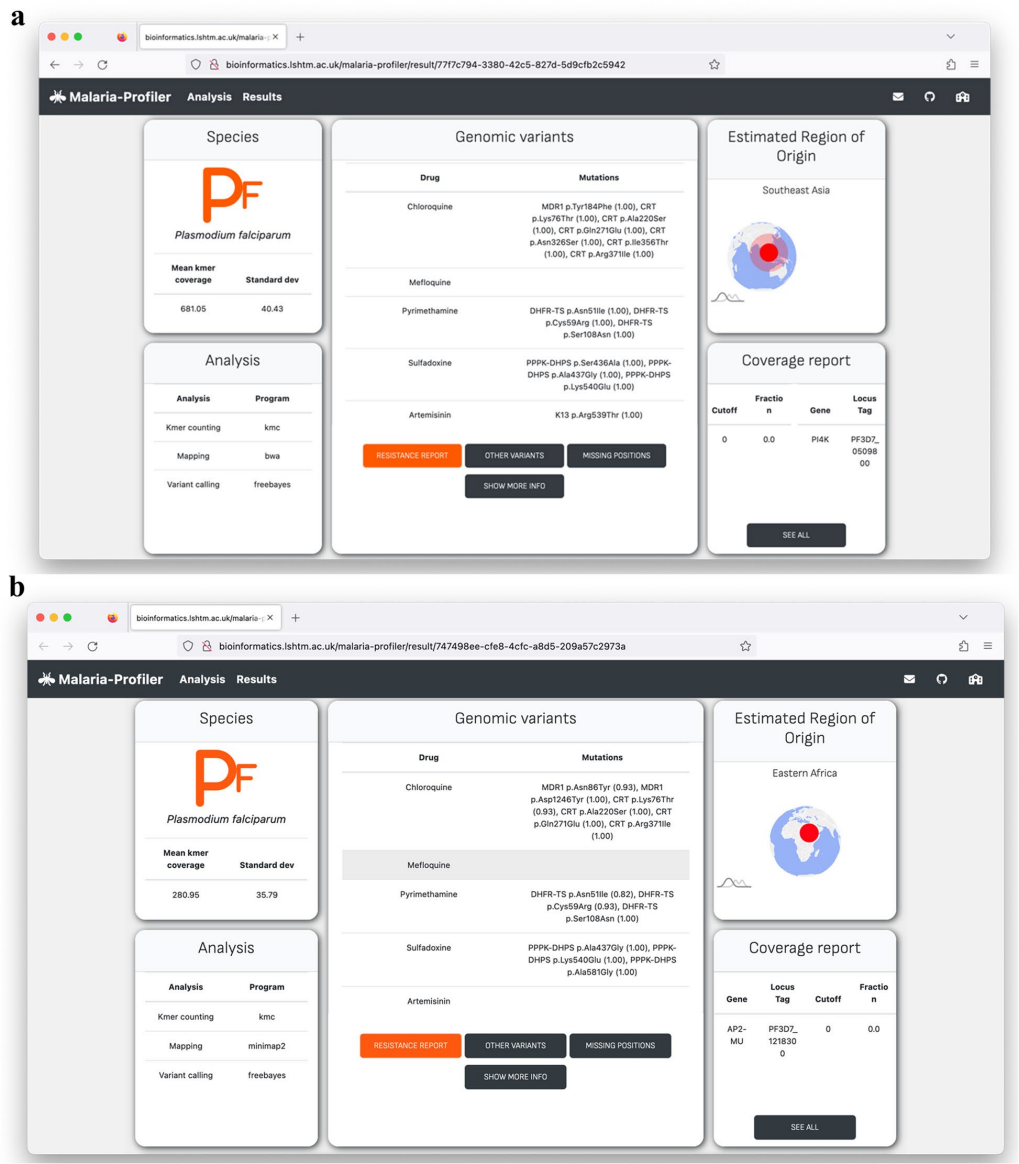
Example of Malaria-Profiler report outputs. **a** Thai isolate confirmed to be *P. falciparum* from Southeast Asia, with a complex drug resistance profile (accession no. ERR248945). **b** A traveller isolate sequenced on Oxford Nanopore Technology and determined to be from East Africa and with chloroquine, Sulfadoxine and Pyrimethamine resistance (accession no. ERR11254081)

Using the known *P. falciparum* markers for chloroquine, SP and artemisinin, the patterns of predicted genotypic resistance were similar to established patterns. Resistance to pyrimethamine was high across all regions (> 87%), leading to high SP prevalence (> 80%), except in Oceania and South America. Chloroquine resistance was lowest in East and West Africa (< 38%), where the drug was withdrawn as a treatment more than 20 years ago, and there has been some reversion back to wild-type alleles [5, 43]. Mutations linked to (partial) resistance to artemisinin in *P. falciparum* were found in Southeast Asia (30.6%), in keeping with their known emergence and spread from the Greater Mekong region [6, 11]. For one such *P. falciparum* isolate with partial resistance to artemisinin, we show the informative nature of the *Malaria-Profiler* Dashboard output (Fig. 2a). The Thai isolate was sequenced on an Illumina platform (accession no. ERR248945), and *Malaria-Profiler* predicts that it is from Southeast Asia, and has a complex drug resistance profile involving genotypic resistance to chloroquine, SP, and artemisinin (Fig. 2a).

### Profiling using ONT platform data

Isolates were sourced from two travellers attending the Radboud University Medical Center who tested positive for malaria. Isolate DNA (isolate1: ERR11254081, isolate2: ERR11254083) was sequenced on the ONT platform to establish their likely geographical source and genotypic drug resistance. Isolate1 was sequenced twice with 410,115 and 1,168,719 reads mapped in total, leading to a median coverage of 31- and 94-fold, respectively. Across both sequencing runs, all positions in candidate genes used for profiling were covered by at least 10 reads. The profiles resulting from each sequencing run were identical. Resistance to chloroquine was predicted through mutations in *pfmdr1* (Asn86Tyr, Asp1246Tyr) and *pfcrt* (Lys76Thr, Ala220Ser, Gln271Glu, Arg371Ile). Resistance to SP was predicted through mutations in *pfdhfr* (Asn51Ile, Cys59Arg, Ser108Asn) and *pfdhps* (Ala437Gly, Lys540Glu, Ala581Gly). The geographic origin was predicted to be East Africa (Fig. 2b), and consistent with the traveller staying in Uganda. Isolate2 had 1,271,185 mapped reads, leading to 109-fold median coverage and all candidate gene positions covered by at least 10 reads. Resistance to SP was predicted through mutations in *pfdhfr* (Asn51Ile, Ser108Asn) and *pfdhps* (Ala437Gly, Lys540Glu). The traveller had been in Rwanda and India, and the predicted geographic origin was Africa (Additional file 1: Fig. S2b), suggesting that the source of infection was the former.

## Discussion

Advances in WGS technology have expanded a role for genome analysis in the clinical laboratory and field settings. Determining the profile of *Plasmodium* species using WGS will guide elimination strategies, including through the monitoring of important mutations temporally and assessing the extent of mixed infections. The sequencing of DNA from malaria infections with low parasite density will be crucial in pre-elimination settings and is possible through low-cost selective whole genome amplification protocols [18]. We have previously shown the robustness of variant calling tools to detect SNPs, small indels and large deletions from WGS data [9, 26, 44]. As WGS is adopted more widely as a diagnostic tool, there is a need for robust and reliable software tools to rapidly process the vast amounts of data generated. Further, the growing application of third and fourth-generation sequencing platforms (e.g. ONT MinION) and linked cost-effective amplicon-based approaches have driven the need to integrate analysis options for these technologies into profiling tools to support their use in a more automated format than currently available.

The *Malaria-Profiler* framework allows for an adaptive mutation library, where the set of barcoding markers can be extended to cover gaps in our knowledge. As our knowledge of *Plasmodium* drug resistance mechanisms (e.g. *P. vivax* chloroquine resistant loci) and geographical-specific markers (e.g. for *P. malariae* and *P. ovale* ssp.) grows, prediction software must be flexible and allow for customisation of barcoding databases. The generation of informative genomic data will be facilitated through advances in sequencing platforms, including low-cost applications of amplicon-based assays that target candidate genes. Further, ONT platforms can implement “adaptive” sequencing, where it is possible enrich on-target reads through real-time alignment to specified genomes of interest and eject uninteresting reads, thereby minimising the generation of contaminant sequences in a clinical sample. Whilst human contaminants in blood are typically removed through sample processing protocols (e.g. SWGA, leucocyte depletion), *Malaria-Profiler* also filters non-*Plasmodium* sequences using bioinformatic methods. Ultimately, if there is insufficient sequence coverage of *Plasmodium* parasite DNA, then *Malaria-Profiler* cannot call variants robustly. A future extension of the software could be to identify potentially informative markers in the human genome [45], such as sickle cell HbS, but this would require extensive evaluation of sequencing protocols and data generated across a range of asymptomatic and clinical blood samples. The increased deployment and availability of such technologies could lead to assessments of *Plasmodium* genetic diversity in sites with currently limited data and studies. There is a constant need to update, re-evaluate and improve mutation libraries in response to new genomic data and functional evidence, including through the implementation of artificial intelligence approaches [36, 46]. To minimise the risk that mutation libraries become unmaintained and remain static versions of evidence at the time, we have hosted the library on a repository that facilitates user input (https://github.com/jodyphelan/malaria-db). Further improvements can involve the exploration of structural changes, such as copy number variants, as some have been linked with the drug resistance. For example, markers of resistance to mefloquine and piperaquine, include amplifications of *Pfmdr1* and *PfPlasmepsin2/3*, respectively. Similarly, low-density infections, and deletions of the *P. falciparum hrp2/3* genes (encoding the HRP2 and HRP3 proteins) [47] present challenges for some rapid diagnostic tests, therefore such deletions could also be included [48]. Whilst analyses of gene coverage are possible through sequencing-based approaches, leading to insights into amplifications and deletions, there may also be SNPs that tag structural variants to facilitate implementation.

In summary, monitoring genetic markers of resistance can help guide antimalarial therapy and surveillance activities. The introduction of drug resistance markers to new geographical areas may be detected through the WGS of clinical samples and analysis of data using *Malaria-Profiler*. Routine WGS across time and geographical regions can detect the presence and spread of established or new markers, and inform infection control practice. WGS has the potential to improve the resolution and timeliness of *Plasmodium* profiling and, in combination with clinical trials and robust experimental work using *Plasmodium* culture and CRISPR-Cas9 systems [49], can lead to new insights into drug resistance mechanisms. *Malaria-Profiler* is a flexible software tool that allows users to rapidly obtain useful information from WGS (and amplicon) data generated by Illumina and MinION platforms to predict species, drug resistance and geographical profiles with high accuracy.

## Conclusions

We have developed an online software tool and methodology that provides rapid analysis of genome sequence data to describe *Plasmodium* species and geographical source and predict resistance to antimalarial drugs. The tool utilises a library consisting of ~ 250 mutations that is the most comprehensive and accurate such data source yet reported. The ability to rapidly analyse raw sequence data and extract information of clinical relevance has advantages over current in vitro drug assays, which require parasite culture-based systems [50]. Accelerated access to tailored treatment could improve cure rates and reduce exposure to ineffective drugs, thereby improving the patient experience and facilitating compliance. The analytical methodology described is customisable to allow moderation of the library to encompass novel mutations and incorporate new drugs should the need arise. Overall, we have shown that *Malaria-Profiler* can be used to reliably predict *Plasmodium* species, geographical source, and drug resistance from WGS. This pipeline can be applied to data from multiple sequencing platforms and can support informatically the application of WGS as a diagnostic and surveillance tool.

### Supplementary Information

**Additional file 1: Fig. S1. **Schematic highlighting the main steps in the Malaria-Profiler pipeline.** Fig. S2. **Malaria-Profiler tool.

## Data Availability

The *Malaria-Profiler* webtool and source code can be accessed (https://github.com/jodyphelan/Malaria-Profiler). The barcoding mutations for speciation, geography and resistance can be accessed (https://github.com/jodyphelan/malaria-db). The raw sequencing data analysed during the current study are available from the ENA and NCBI databases. The Pf6 database was used for *P. falciparum* (https://www.malariagen.net/apps/pf6; [51]), and the Pv4 database was used for *P. vivax* (https://www.malariagen.net/data/open-dataset-plasmodium-vivax-v4.0; [52]). For *P. knowlesi*, the raw sequence data is available from the ENA (accession numbers: PRJEB10288, PRJEB1405, PRJEB23813, PRJEB28192, PRJEB33025, and PRJNA294104) [17, 20]. Similarly, raw sequence data are available for *P. malariae* (accession number: PRJEB33837) [19] and *P. ovale* ssp (accession number: PRJEB51041) [19]. Raw sequence data for the two newly sequenced *P. falciparum* isolates are available from the ENA (https://www.ebi.ac.uk/ena/browser/view/ERR11254081; www.ebi.ac.uk/ena/browser/view/ERR11254083).

## Abbreviations

SP: Sulfadoxine-pyrimethamine
WGS: Whole genome sequencing
WHO: World Health Organization

## Availability of data and materials

Project name: *Malaria-Profiler.*

Project home page: https://github.com/jodyphelan/Malaria-Profiler; https://github.com/jodyphelan/malaria-db.

Programming language: Python.

Operating system(s): Platform independent.

Other requirements: None.

License: GNU GPL.

Any restrictions to use by non-academics: None.
