# Supplementary material for: Rapid profiling of Plasmodium parasites from genome sequences to assist malaria control
Source: Genome Med. 2023 Nov 10;15:96. doi: 10.1186/s13073-023-01247-7 (PMC10636944; doi:10.1186/s13073-023-01247-7)

Additional file 1

Fig. S1

Schematic highlighting the main steps in the *Malaria-Profiler* pipeline. The first step entails finding k-mers that are species specific using *kmc* software. The second step maps the reads to the reference genome using either *BWA-mem* software for Illumina or the *minimap2* tool for ONT and stores the alignments in a bam file. Additionally, Illumina reads are first trimmed using *trimmomatic* software. The third step calls variants from the bam alignment file using *freebayes* software. These are then compared to known resistance variants to predict drug resistance. Orange icons indicate files, blue icons indicate software processes and green icons indicate databases.

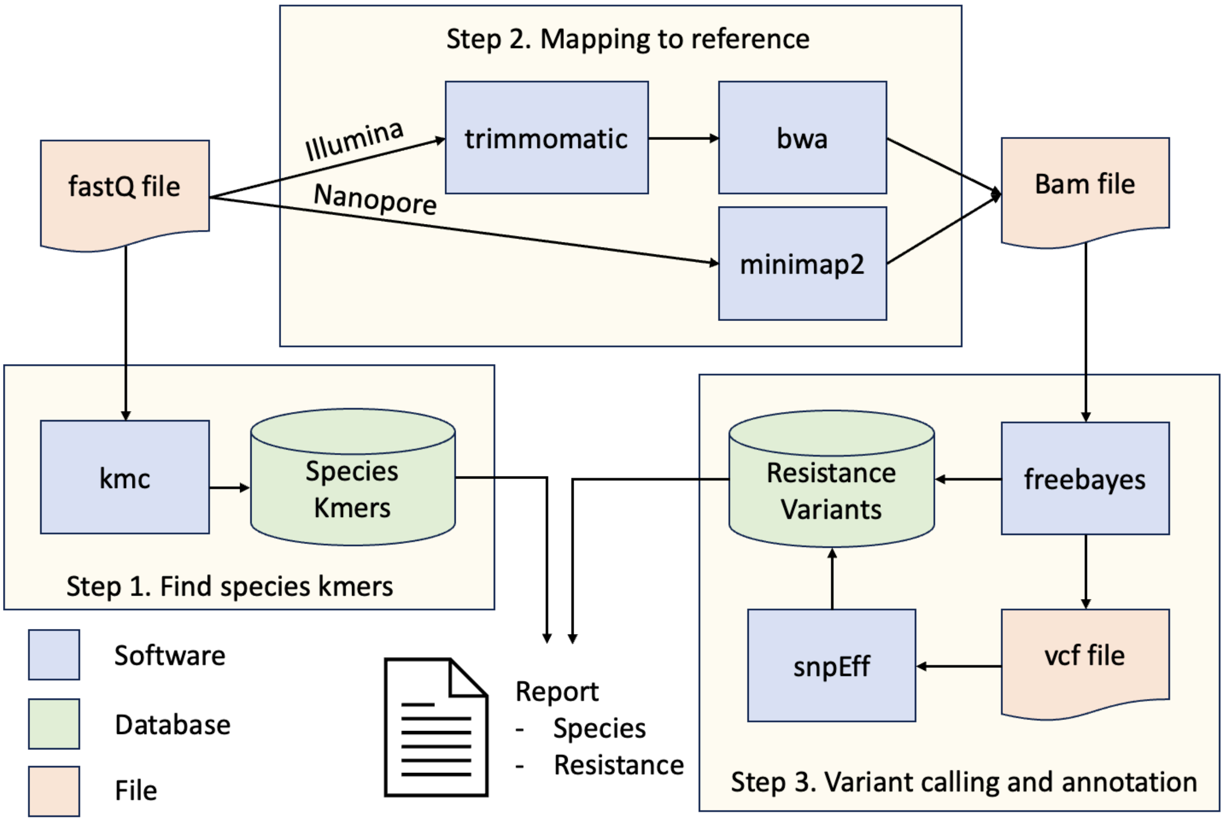

Fig. S2

Malaria-Profiler tool

(a) The website for data input (bioinformatics.lshtm.ac.uk/malaria-profiler)

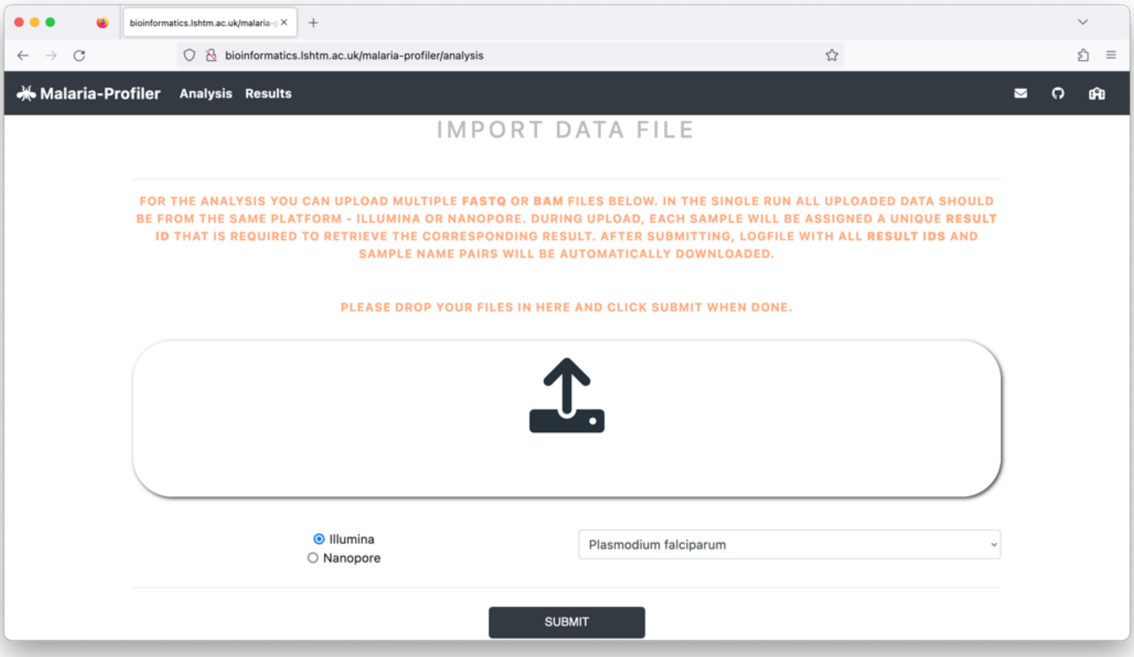

(b) Example of an isolate from a traveller diagnosed and treated with malaria in Europe, sequenced on Oxford Nanopore Technology (ONT), and determined to be from Africa and resistant to Sulfadoxine and Pyrimethamine (SP) (accession no. ERR11254083).

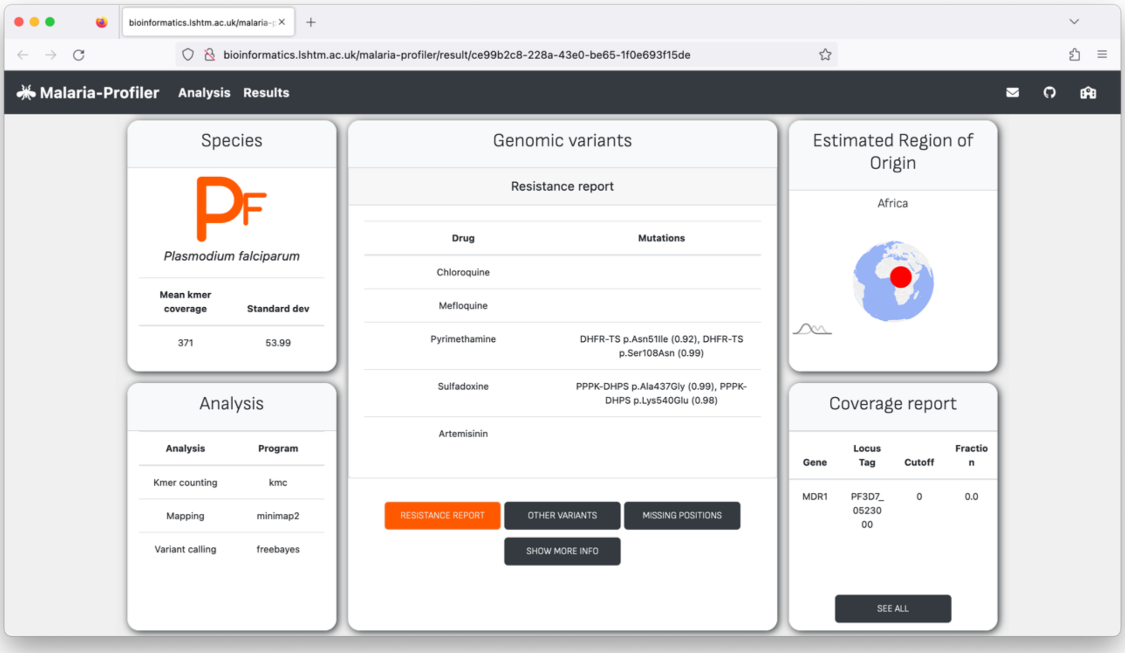

Supplement: Supplementary file 1 — Additional file 1: Fig. S1. Schematic highlighting the main steps in the Malaria-Profiler pipeline. Fig. S2. Malaria-Profiler tool. [file 13073_2023_1247_MOESM1_ESM.pdf]
